# Cerebrovascular and Neurological Disorders: Protective Role of NRF2

**DOI:** 10.3390/ijms20143433

**Published:** 2019-07-12

**Authors:** Farzane Sivandzade, Aditya Bhalerao, Luca Cucullo

**Affiliations:** 1 Department of Pharmaceutical Sciences, Texas Tech University Health Sciences Center, Amarillo, TX 79106, USA; 2 Center for Blood Brain Barrier Research, Texas Tech University Health Sciences Center, Amarillo, TX 79106, USA

**Keywords:** oxidative stress, pathway, NRF2, cerebrovascular, neurodegenerative, alternative

## Abstract

Cellular defense mechanisms, intracellular signaling, and physiological functions are regulated by electrophiles and reactive oxygen species (ROS). Recent works strongly considered imbalanced ROS and electrophile overabundance as the leading cause of cellular and tissue damage, whereas oxidative stress (OS) plays a crucial role for the onset and progression of major cerebrovascular and neurodegenerative pathologies. These include Alzheimer’s disease (AD), Parkinson’s disease (PD), amyotrophic lateral sclerosis (ALS), Huntington’s disease (HD), stroke, and aging. Nuclear factor erythroid 2-related factor (NRF2) is the major modulator of the xenobiotic-activated receptor (XAR) and is accountable for activating the antioxidative response elements (ARE)-pathway modulating the detoxification and antioxidative responses of the cells. NRF2 activity, however, is also implicated in carcinogenesis protection, stem cells regulation, anti-inflammation, anti-aging, and so forth. Herein, we briefly describe the NRF2–ARE pathway and provide a review analysis of its functioning and system integration as well as its role in major CNS disorders. We also discuss NRF2-based therapeutic approaches for the treatment of neurodegenerative and cerebrovascular disorders.

## 1. Introduction

Oxidative stress (OS), one of the main indications of various pathologic processes, results from the production of ROS including hydrogen peroxide, superoxide, and hydroxyl free radicals. In turn, these highly reactive compounds promote lipid peroxidation, protein backbone fragmentation, genotoxicity, mitochondrial depolarization, and apoptosis that subsequently cause serious damage to tissues and organs including the brain [1,2,3]. The majority of this excessive reactive oxidative species is expected to be produced by oxidative phosphorylation responses in the mitochondria [4,5]. Cells have developed several defense mechanisms equipped with the capacity to upregulate the expression levels of cytoprotective enzyme genes in order to scavenge free radicals and reduce the risk for the cellular damaging effect of ROS [6,7]. Nuclear factor erythroid 2-related factor (NRF2) is one of the most important transcription factors that enhance the expression of antioxidant reaction to oxidant stress [5].

NRF2 (a member of the Cap-n-Collar family of basic leucine zipper proteins) as a ubiquitously expressed redox-sensitive transcription factor, primarily modulates the activation of biological systems encompassing anti-inflammatory molecules, antioxidants (such as thioredoxin, glutathione, and others), phase I and II drug metabolizing enzymes (such as cytochrome P450s), phase III enzymes (efflux transporters), and free radical scavengers [6,8,9,10,11,12]. Consequently, cell vulnerability to the detrimental effects of ROS and OS damage to mitochondrial function (leading to cell apoptosis) are enhanced by downregulation or suppression of NRF2 activity [13,14,15,16]. Cellular OS initiates a sequence of biological responses so that NRF2 (residing in the cytoplasm at a low basal level) translocates into the nucleus [17] where it forms a heterodimer with small Maf proteins (MafG, MafK, MafF). Coupling with Mafs endows NRF2 with a DNA-linking capacity to bind to the ARE sequence and initiate the transcription of ROS detoxification genes [18].

Due to the fact that NRF2 is likely to control, modulate, and sustain the expression of detoxification and antioxidative response elements and other types of protective elements (which include the ubiquitary iron exporter ferroportin 1, anti-apoptotic B-cell lymphoma 2, brain-derived neurotrophic factors, the peroxisome proliferator-activated receptor gamma coactivator 1-alpha (PGC-1α), the mitochondrial-nuclear respiratory factor 1 (Nrf1), and the autophagic protein p62) [19,20,21,22,23,24,25], its activation plays a considerable role in counteracting acute injuries, effects of xenobiotics, inflammation, and many other stimuli that are promoted by OS [17,26]. With regard to OS being implicated in several pathologies, current research is focused on pathogenic mechanisms that lead to mitochondrial dysfunction and redox imbalance [26]. In this review, we provide a detailed analysis of the current understanding of the NRF2–ARE system and its role in major CNS disorders. We also include NRF2-focused therapeutic approaches for the treatment of cerebrovascular and neurodegenerative diseases.

## 2. NRF2 Regulation and Response to Oxidative Stress

Domain analysis by high-resolution crystal structure and nuclear magnetic resonance spectroscopy has shown that the molecular structure of NRF2 includes seven functional domains (Neh1–Neh7) that regulate its transcriptional activity and stability [27]. The first conserved domain, Neh1, containing basic bZIP motif binds, to the ARE sequence exposing a nuclear localization signal required for translocation of released NRF2 from Kelch-like ECH-associated protein 1 (KEAP1) into the nucleus [28,29,30]. Neh1 and Neh2 play differing roles with respect to NRF2 regulation. While Neh1 modulates NRF2 protein stability through interaction with the E2 ubiquitin-conjugating enzyme, Neh2, a negative regulatory domain located in the N-terminal region, promotes NRF2 ubiquitination followed by proteasomal degradation, which is a result of increased KEAP1–NRF2 binding [31]. While Neh3 (which is located in the carboxyl-terminal region of the protein) modulates the transcriptional activation of the ARE genes [27,32], the Neh4 and Neh5 domains play a cooperative role in facilitating NRF2 transcription by binding to a transcriptional co-activator [33] and also increases NRF2–ARE gene expression by interfacing with the nuclear cofactor RAC3/AIB1/SRC-3 [33,34]. The Neh6 and Neh7 domains control KEAP1-independent degradation of NRF2 and regulate the activity of NRF2 so that KEAP1-alternative pathway of NRF2 degradation arises based on the recognition of phosphorylated Neh6 by the E3 ligase adapter beta-TrCP [35,36,37] and Neh7 inhibits NRF2 via interaction with retinoid X receptor α [38]. The main step in detoxification is the nuclear and cytoplasmic disposition of NRF2 so that under basal conditions, NRF2 is rapidly polyubiquitinated while cellular redox homeostasis is sustained by the accumulation of the NRF2 in the nucleus to mediate the normal expression of ARE-dependent genes [26,39]. 

KEAP1, the main intracellular regulator of NRF2, is composed of three main domains (totaling 624 amino acids) including the Broad-complex (1), Tramtrack (2), and the Bric-a-Brac (BTB) domain (3) which includes a cysteine-rich region and a double glycine repeat -DGR- binding site between KEAP1 and NRF2. Several cysteine residues within the BTB domain act as OS sensors and/or inducer ligands within the cell’s environment [27]. The activity of the NRF2–ARE signaling pathway is controlled by degradation and sequestration of NRF2 in the cytoplasm through binding with KEAP1 [5,26]. Other factors including post-transcription changes, gene polymorphisms in the promoter region, and protein–protein interactions are also influenced by NRF2 basal activity [39,40]. It is noteworthy that in response to mitochondrial oxidative stressors NRF2 provides direct interaction with the mitochondrial membrane [41].

Under basal conditions, KEAP1 binds to NRF2 in the cytoplasm and enhances the ubiquitination and proteasomal degradation of NRF2, whereas in response to oxidative stress condition, the NRF2 DLG motif is released from the DGR domain in KEAP1, which then undergoes conformational changes and dissociate NRF2 from itself to shift into the nucleus freely [39]. Independently from KEAP1 activity, not only phosphorylation of NRF2’s serine enhance separation of NRF2 from KEAP1 [26], but also glycogen synthase kinase-3β (GSK-3β), synthesis of specific microRNAs, methylation of CpG islands, histone phosphorylation, and acetylation modulate the expression and activation of NRF2 [6,42,43,44]. This cytoprotective pathway encompasses detoxification systems such as oxidation/reduction factors (Phase I), conjugation enzymes (Phase II), and drug efflux transporters (Phase III) [39,44]. Phase I includes more than 500 products encompassing genes encoding proteins for redox balancing factors, stress response proteins, detoxifying enzymes, and metabolic enzymes such as NAD(P)H:quinone oxidoreductase 1 (NQO1), heme oxygenase-1 (HO-1), superoxide dismutase (SOD), glutathione S-transferase (GST), glutathione reductase (GSR), glutathione peroxidase (GSH-Px), carbonyl reductase (CR), and glutamate-cysteine ligase (GCL) [45,46]. Metabolites then undergo Phase II metabolism where they are conjugated with large polar groups (such as glucuronic acid, glutathione, sulfate, or glycine) to increase their water solubility and facilitate their excretion. Thus, it seems that NRF2 plays a major role in modulating/promoting the process of detoxification [5,30]. NRF2 also controls/enhances the expression of active efflux transporters that remove or keep out potentially detrimental endogenous or xenobiotics of the cell [39] as well as tight junction (TJ) expression and BBB integrity at the neurovascular unit [47]. NRF2 nuclear accumulation can also have harmful effects [29] which accounts for the necessity of autoregulating the cellular levels of NRF2 [32,48]. For this end, the cullin3/ring box 1 (Cul3/Rbx1) E3 ubiquitin ligase complex promotes polyubiquitination of NRF2 coupled with KEAP 1 followed by NRF2 proteasomal degradation. This mechanism controls the “switching off” of NRF2-activated gene expression in the nucleus [32]. 

## 3. Crosstalk between NRF2 and Other Regulatory Signaling Pathways

According to numerous studies, there is a crosstalk between NRF2, KEAP1, and other signaling pathways. In the KEAP1-independent degradation pathway mediated by the cullin3/ring box 1 (Cul3/Rbx1) E3 ubiquitin ligase complex, NRF2 acts as a regulator for the complex. This occurs through NRF2 binding to the KEAP1 region via the Neh2 domain, which enhances NRF2 polyubiquitination and then degradation by the 26S proteasome [1,26,35]. Moreover, this Cul3/Rbx1-mediated degradation of NRF2 is controlled by phosphorylation of the Neh6 domain of NRF2 which is regulated by GSK-3 [37]. Furthermore, deletion of PTEN (phosphatase and tensin homolog) deleted from chromosome10 promotes NRF2 accumulation in KEAP1-deficient liver, suggesting that the inactivation of GSK-3 and the activation of the PI3K-Akt pathway by the loss of PTEN lead to the inhibition of NRF2 degradation via the Cul1/Rbx1 pathway [7,49].

Another essential crosstalk occurs between the KEAP1–NRF2 system and the cellular autophagy pathway. Autophagy-adaptor protein p62 competes with KEAP1 for binding with NRF2 using the STGE motif so that p62 promotes the stabilization of NRF2 and the upregulation of NRF2 activity through this competition [24]. Interestingly, phosphorylation of the STGE motif in p62 causes enhanced binding affinity to KEAP1 [50]. A considerable finding is that NRF2 is predominantly degraded through the proteasome pathway, whereas KEAP1 is degraded through the autophagy pathway maintaining the integrity and homeostasis of the KEAP1–NRF2 system by governing KEAP1 turnover [51]. Thus, the KEAP1–NRF2 system is regulated through the two significant cellular proteolysis pathways.

**Figure 1:**
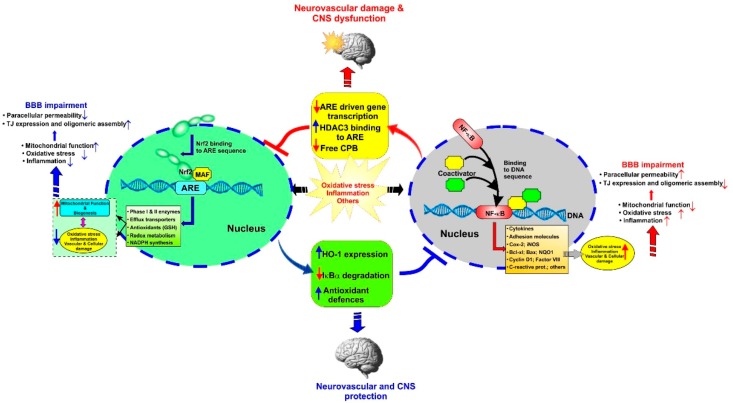
Schematic representation of the NRF2–NF-kB cross talk with respect to oxidative stress and inflammatory stimuli. Note that an “up arrow” indicates upregulation while a “down arrow” indicates downregulation.

NF-κB is a rapid response factor to harmful cellular stimuli which is associated with Inhibitor of κB (IκB) proteins (which include IκBα, IκBβ, IκBε, and Bcl-3 although IκBα is the major IκB protein). In response to noxious stimuli (such as OS and pro-inflammatory cytokines like TNF-α), the IκB proteins (which under normal conditions mask the nuclear localization signals—NLS—of NF-κB and keep this factor sequestered in an inactive state in the cytoplasm) undergo phosphorylation by the IκB kinase complex, followed by ubiquitination, and proteasomal degradation [5]. Degradation of IκB frees NF-κB can then translocate into the nucleus, and alter (promote) the transcription of genes responsible for both the innate and adaptive immune responses [52]. Recent findings have confirmed the crosstalk between NRF2 and NF-κB signaling pathways under OS conditions as well as a variety of pathophysiological conditions [53] in us much that pro-inflammatory and immune responses promoted by the activity of NF-κB are contrasted by upregulation and activation of NRF2 [5]. By contrast, enhanced inflammation could be associated with downregulation of NRF2 activity (see Figure 1). 

In a study on NRF2 knock out mice, hypersensitiveness to the inflammation induced by LPS was observed while these harmful effects were repealed following treatment with sulforaphane as a NRF2-dependent gene expression enhancer [54]. Although several studies on other cell types reported similar observations, the interconnection of NRF2 and NF-κB to coordinate anti-oxidative and inflammatory responses is not completely understood [55,56,57,58,59,60]. In a study by Cuadrado et al., the investigators observed that upregulation of RAC1 (a pleiotropic a small G-protein of the Rho family and a modulator of NF-κB transcription) activated the inflammatory pathway through an interplay between NF-κB and NRF2 [61] so that RAC1 participated in NADPH oxidase-dependent production of superoxide, whereas activation of NF-κB was mediated through RAC1 dependent ROS formation [62]. In fact, NRF2 deletion promoted the activation of NF-κB dependent inflammatory markers, while NRF2 overexpression inhibited RAC1-dependent activation of NF-κB pathway, suggesting that RAC1 affects NRF2 and NF-κB. 

## 4. Role of NRF2 in Aging and Traumatic Brain Injury

Non-effective antioxidative responses to excessive ROS production and changes in redox signaling playing are one of the major reasons for advanced aging, whereas the inability to properly counteract OS leads to the progressive accumulation of OS-induced cellular damage [63,64]. Recent studies have, in fact, shown that age-related OS damages are dependent upon decreased antioxidant responses, as well as proteasome reduction, and reduced efficiency of mitochondrial proteases. The resulting effect of this impaired antioxidative response and inability to effectively maintain the redox balance is an accumulation of intracellular and intramitochondrial aggregates of oxidized proteins [65]. Similarly, the decreased level NADPH and GSH with aging could likely be due to a decreased cellular antioxidative capacity, as well as reduced intake of dietary antioxidants. Although there is a lot of dispute over the effect of age on the basal expression levels of antioxidative response factors, it seems that the primary underlying cause is a reduced NRF2 activity [66,67,68].

In respect to traumatic brain injury (TBI), disruption in the normal brain function following TBI is one of the foremost causes of death as well as severe emotional, physical, and cognitive impairments [69,70,71]. In spite of the pathogenic role of the primary brain injury immediate to TBI, the post-traumatic secondary injury derived from OS, inflammation, excitotoxicity, enhanced vascular permeability, and BBB impairment can significantly worsen post-traumatic brain damage as well as clinical outcomes [72,73]. Excessive ROS generation following cell damage, neuronal cell death, and brain dysfunction are the results of several secondary biochemical and metabolic changes in the cells [74]. According to recent studies, NRF2 plays a neuroprotective role in TBI so that NRF2 activation counteracts TBI-induced OS, loss of BBB integrity, etc. Unsurprisingly, impairments of the NRF2–ARE pathway leading to reduced activity of this protective system can result in more extensive post-TBI tissue damage, thus aggravating the secondary injury and worsening outcome. Accordingly, promoting upregulation of NRF2 activity could be exploited to reduce post-traumatic brain injuries, improve clinical outcomes, and reduce the risk of additional neurological disorders [75,76].

## 5. Ischemic Stroke and Protective Role of NRF2 

Stroke, the fifth leading cause of death in the United States and a major cause of permanent disability, is defined by a bursting or blockage of blood vessels resulting in the sudden interruption of the local blood supply and the initiation of an anoxic and hypoglycemic state in the affected brain tissue [4]. Moreover, neuronal cell membrane depolarization causes the release of the neurotransmitter glutamate, which is the activator of the ionotropic glutamate receptor *N*-methyl-d-aspartate (NMDA) [5]. The resulting opening of these non-selective cation channels leads to calcium overload and neuronal cell death [77]. These events are associated with excessive ROS production by the mitochondria which overwhelm the antioxidant defenses, leading to post-ischemic inflammation and enhanced brain tissue damage [78,79,80,81]. Furthermore, degradation of the structural proteins of the vascular wall and loss of BBB integrity also occur as a result of blood flow restoration, which suddenly enhances tissue oxygenation further exacerbating ROS production, inflammatory responses, and OS damage [5]. Adhesion of leukocytes across the blood vessels and transmigration into the brain parenchyma is facilitated by the concurrent expression of vascular adhesion molecules on the luminal surface of the vascular walls. Based on these premises, it is evident that control of ROS levels and OS prevention could be a potential therapeutic strategy to address post-ischemic secondary brain injury and improve stroke outcome [5,82,83,84]. Recent studies demonstrated that the protective effect of interactions between p62 and the NRF2–EpRE signaling pathway inhibited OS damage during cerebral ischemia/ reperfusion in rat undergoing transient middle artery occlusion (tMCAO) and also promoted NRF2 activity to lower the infarct volume and post-ischemic neurocognitive impairments [85,86]. NRF2 activity is also crucial to protect the brain against injury. In fact, NRF2 activation through the use of pharmacological enhancers improved neuronal cell viability, decreased BBB permeability, and promoted the transcription of cytoprotective genes [87,88]. Furthermore, enhanced infarct size, inflammatory damages, and neurological deficits were reported in NRF2 KO mice when compared to controls (wild-type mice). By contrast to controls, the use of NRF2 enhancer in knock out mice did not elicit any beneficial effect [89]. Most recently, other studies have shown that NRF2 downregulated the activity of the NOD-like receptor protein 3 (NLRP3) inflammasome by acting on thioredoxin-1 (Trx1)/thioredoxin interacting protein (TXNIP) complex [82]. The NLRP3 inflammasome plays a key role in inflammation damage in cerebral ischemia-reperfusion injury by promoting the activity of interleukin-23/interleukin-17 axis which contributes to the ischemic reperfusion damage at the CNS [90]. The activation of NLRP3 is dependent upon the interaction with TXNIP, which dissociates from the Trx1/TXNIP complex under OS. Thus, it is clear how targeting NRF2 represent a viable target for the treatment of ischemia and reperfusion injury.

## 6. Role of NRF2 in Neurodegenerative Diseases

**Figure 2:**
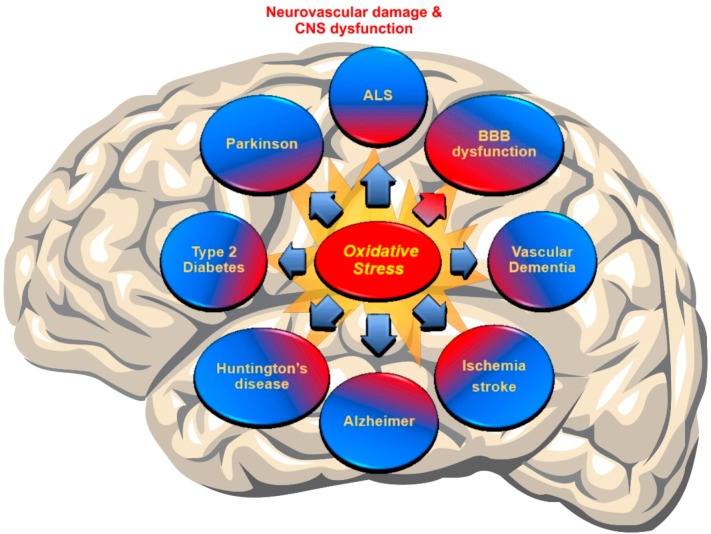
Schematic representation of the Cerebrovascular and neurodegenerative diseases associated with impaired redox metabolism and oxidative stress.

Recent discoveries have mentioned OS as a major pathogenesis of neurodegenerative disorders (NDDs) due to the accumulation of ROS [4,91]. In fact, a failure in maintaining the proper balance between ROS generation and their neutralization causes a disruption of brain homeostasis leading to neurodegenerative disorders [92,93] (see Figure 2).

As commonly observed in neurodegenerative disorders, including Alzheimer’s disease (AD), Parkinson’s disease (PD), amyotrophic lateral sclerosis (ALS) and Huntington’s disease (HD), ROS production overwhelms the antioxidative response system causing cellular damage so that elevated levels of oxidative markers and damaged cell components have been diagnosed in patients with neurodegenerative diseases [94]. In spite of the poor understanding of the underlying mechanisms linking NRF2 with the onset/progression of these disorders, proteinopathies are the pathogenic hallmark shared between these disorders [6]. The impairment of mitochondrial function is another common feature of these neurodegenerative disorders and also promotes ROS generation, ATP depletion, and inflammation [6,51,95,96,97,98]. Thus, this concept could provide opportunities for interventions focused on restoring/normalizing the cellular antioxidative response and decreasing inflammation and following the reduced progression of these diseases. 

Alzheimer’s disease: A neuropathological hallmark of AD is the formation of intracellular neurofibrillary tangles (NFTs) and extracellular senile plaques (SPs) composed of small Aβ peptides [99]. Several studies propose that OS is an early prodromal event for progressive neurodegenerative disorders [100]. According to several studies on AD, NRF2 was able to provide a neuroprotective effect by decreasing ROS generation and ROS-induced toxicity mediated by Aβ [101,102]. Supporting data have shown that NRF2 activators, such as sulforaphane (SFN) lower toxin-induced Aβ1-42 secretion, while enhancing cell viability and improving cognitive function [103,104]. These beneficial effects may be due to the formation of Aβ aggregates or the inhibition of the release of monomer/oligomeric Aβ from dead cells [105]. A recent study also outlined the role of NRF2 in facilitating autophagy as well as altering β-Amyloid precursor proteins (APP) and Aβ processing whereas NRF2 knockout APP/PS1 mice showed increased accumulation of insoluble APP fragments and Aβ as well as mammalian targets of rapamycin (mTOR) activity [5,106]. The investigators also found that overexpression of mitochondria catalase in APP transgenic mice (Tg2576), decreases the formation of full-length APPs and lowers soluble and insoluble Aβ levels. From a clinical perspective, this may translate into extending the lifespan of the patient while improving working memory [5]. In a recent study, Rojo et al. demonstrated the protective effect of NRF2 against exacerbation of astrogliosis and microgliosis using transgenic mouse models [105]. Specifically, the investigators have shown a reduction in homeostatic responses with aging along with NRF2 activity resulting in reduced protection against proteotoxic, inflammatory and oxidative stress stimuli [5,107]. 

Parkinson’s disease: PD is a progressive neurodegenerative disorder characterized by lowered dopamine levels in the striatum due to the loss of dopaminergic neurons located in the *substantia nigra* affecting movement [94]. The initial symptoms in PD patients sometimes are tremors affecting one hand or slowing of movement. With the progression of the disease controls over movement is completely compromised and the effects are extended to neurocognitive functions dementia [5]. The certain diagnosis of PD in both familial and sporadic PD patients is the presence of Lewy bodies (LBs) as abnormal protein aggregates developing inside nerve cells. The main constituent of LBs is Alpha-synuclein (αSyn) which is a small protein with 140 amino acids. αSyn is abundant in presynaptic nerve terminals playing a role in synaptic transmission and dopamine levels adjustment [5]. Recent studies strongly postulate the association between PD with abnormal ROS production promoted by the dopamine metabolism, excitatory amino acids and iron content [100]. Moreover, it is emphasized that this increased OS plays a pivotal role in αSyn proteostasis, whereas NRF2 activity can counteract αSyn production and the associated cellular damage [101,108,109,110,111]. Recently, NRF2 overexpression has not only confirmed the reduction of the generation of αSyn aggregates in the CNS [112], but also the activation of NRF2 has appeared to prevent the loss of dopaminergic neurons mediated by αSyn and the consequent impairment of motor functions [5,113]. NRF2 deficiency and promoted expression of αSyn experienced enhanced loss of dopaminergic neuron and increased neuroinflammation and protein aggregation, whereas the enhanced expression level of NRF2 in a mutant αSyn transgenic mouse model, provided neuroprotective effects [5,57,111].

Huntington’s disease: HD as an inherited neurodegenerative disease is characterized by the loss of GABAergic inhibitory spiny projection neurons in the striatum [94] due to abnormally elongated poly-glutamine (polyQ) stretch encoded by the atypical expansion of adenine, cytosine, and guanine (CAG) trinucleotide repeats at the huntingtin protein (Htt). According to several in vitro studies, NRF2 activation can play a protective role in the reduction of mHtt-induced toxicity, while in HD patients the initiation of the NRF2–ARE system in striatal cells in response to OS failed because of the concurrent activation of the autophagy pathway [114,115]. Moreover, additional data have confirmed that Htt aggregation directly enhanced ROS generation promoting cell toxicity [116]. Furthermore, co-transfection of NRF2 with mHtt in primary striatal neurons, reduction of the mean lifetime of mHtt N-terminal fragments, and, subsequently, improvement of cell viability suggest that NRF2 is more likely to decrease mHtt -toxicity by negatively affecting its aggregation [117]. 

Amyotrophic lateral sclerosis: ALS is a progressive neurodegenerative disease characterized by the loss of motor neurons in the ventral horn of the spinal cord and in the motor cortex. The disease leads to progressive motor weakness and loss of controls of voluntary movements [5,94]. Although, for more than two decades, the mutation of Cu–Zn superoxide dismutase 1 (SOD1) was the only genetic aberration relevant to the initiation of familial ALS, recent studies have found more abnormalities associated with the onset of sporadic and non-SOD1 familial ALS, including a host of RNA/DNA-binding proteins such as the 43-kDa transactive response (TAR) DNA-binding protein (TDP-43) and the fused in sarcoma/translocated in liposarcoma (FUS/TLS) [5]. Several recent studies support that NRF2 activation plays a protective role against OS and cell death promoted by the SOD1 mutant protein so that glial NRF2 overexpression improves the survival of the spinal cord’s motor neurons and extends their viable lifespan [118,119]. Additional studies will be required to evaluate the impact of NRF2 on cellular proteostasis as well as other ALS-associated gene mutations and the effect of NRF2 stimulation on late-stage microglia activation to prevent OS.

## 7. Role of NRF2 in Blood-Brain Barrier (BBB) Integrity and Function 

In the central nervous system, the vascular endothelium acquires a set of specific characteristics and functions that differ from other vascular beds [120]. This specialized endothelium, which forms the BBB, becomes a dynamic functional interface between the blood and the brain that strictly regulates the passage of substances, maintains the brain homeostasis, and protects the brain from pathogens as well as endogenous and xenobiotic substances [69]. According to numerous studies, there is a relationship between NRF2 and BBB relevant to cerebrovascular disorders, so that NRF2 signaling plays a neurovascular protective role in the conservation of the BBB and CNS [8,11,121,122]. With regard to BBB endothelium, it has been emphasized that NRF2 upregulates the expression of tight junctional proteins (TJ), promotes redox metabolic functions, and produces ATP with mitochondrial biogenesis [3,11,122,123]. In fact, recently published data from side by side experiments investigating the impact of electronic cigarettes (e-Cig) versus TS on mouse primary brain microvascular endothelial cells (BMVEC) clearly showed that NRF2 was strongly activated by the resulting OS and promoted upregulation of its downstream signaling molecule NQO-1 [122], whereas NQO-1 exerts acute detoxification and cytoprotective functions. However, chronic exposure to these pro-oxidative stimuli ended up compromising NRF2 activity and that of its downstream effector NQO-1. These resulted in an overall impairment of BBB integrity associated with increased permeability to paracellular markers and decreased trans-endothelial electrical resistance (TEER) [83,122]. In addition to the loss of BBB integrity, in vivo data also showed upregulation of inflammatory markers including vascular adhesion molecules and pro-inflammatory cytokines as well as blood hemostasis changes favoring blood coagulation and, therefore, risk of stroke. Recent preliminary data and work by others have also clearly demonstrated that NRF2 modulates mitochondrial biogenesis, redox metabolism, and antioxidant/detoxification functions, thus, strongly suggesting that impairment of NRF2 activity can negatively affect mitochondrial biogenesis and function [3]. Altogether, these studies have shown that NRF2 plays a major role in critical BBB cellular functions ranging from modulation of barrier integrity, inflammatory responses, redox metabolism, and antioxidative responses [4,5,6,44,66,88,105,121,122,124,125]. In fact, cerebrovascular and neurodegenerative disorders such as subarachnoid brain hemorrhage, MS, ALS, AD, PD, stroke, and type-2 diabetes mellitus (TD2M) have been tied to dysfunctions of NRF2 activity [6,11,51,121,126,127,128,129,130]. Unsurprisingly, the activation of the NRF2–ARE system can potentially prevent/reduce the BBB impairments and, consequently, decrease some types of brain injury [131]. Since vascular endothelial dysfunction and consequent CNS damages have been relevant to ROS [132,133,134] and OS-driven inflammation [135], NRF2 activation is likely to preserve the BBB by maintaining ROS homeostasis that ultimately leads to a decrease in the risk of cerebrovascular, neurodegenerative, and CNS disorders [85,131,136,137,138]. For instance, the well-known NRF2 promoter/activator Sulforaphane (SFN) has been shown to have neuroprotective characteristics that counteract oxidative stress by enhancing NRF2 activation [51,131,139,140,141,142,143,144,145,146] and regulating antioxidant reactions [18].

## 8. Role of NRF2 in Tobacco Smoke-Induced Cerebrovascular Disorders 

Cerebrovascular and BBB dysfunction promoted by tobacco smoke (TS) are also associated with the initiation of various neurovascular and neurodegenerative diseases linked to dysregulation of NRF2 activity such as stroke, vascular dementia, and previously noted neurogenerative disorders [39,85,122]. This is not surprising since TS contains over 7000 chemicals including nicotine and various ROS (e.g., H_2_O_2_, epoxides, nitrogen dioxide, peroxynitrite-ONOO^−^, etc.) that cross the lung alveolar wall and raise systemic OS. At the cerebrovascular level, this promotes oxidative damage and BBB breakdown via tight junction (TJ) modification and the activation of proinflammatory pathways [11,147]. Under normal conditions, ROS are scavenged by endogenous antioxidants involving vitamins such as ascorbic acid and α-tocopherol or intracellularly converted into less reactive molecules by superoxide dismutase (SOD), catalase (CAT), and glutathione peroxidase (GSH-Px). However, containing excessive pro-oxidant substances, chronic exposure to active and passive smoking can overwhelm these protective mechanisms. Furthermore, the protective nature of NRF2 may be altered in smokers by means of somatic mutation, epigenetic alteration, and accumulation of disruptor proteins, thereby promoting cell resistance and proliferation of cancerous cells as indicated by other studies [122,148,149]. 

**Figure 3:**
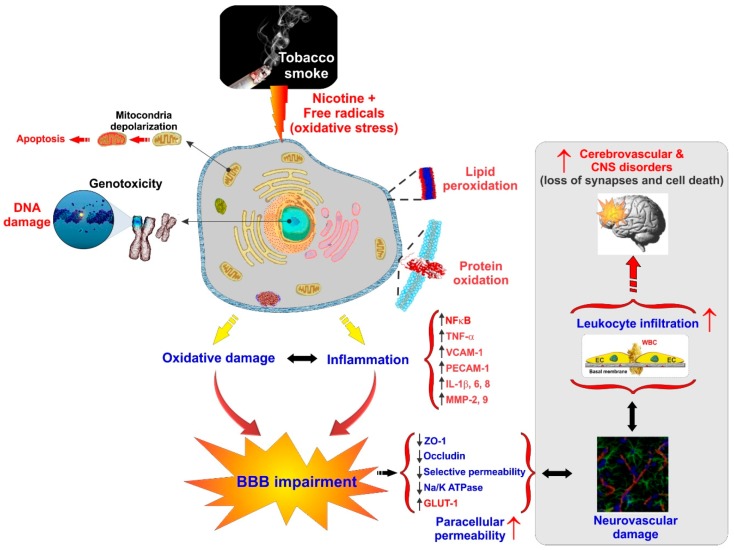
Schematic illustration of the impact of smoking on BBB impairment and the onset of cerebrovascular and CNS disorders. Note that an “up arrow” indicates upregulation while a “down arrow” indicates downregulation.

According to several recent studies, NRF2 enhancers can counteract OS and possibly decrease the burden of neuropathologies including ischemic and cerebral stroke [5,85,150,151,152,153]. Recently Prasad et al. have confirmed the upregulation of NRF2 upon acute TS/e-Cig exposure and its impact on mitochondria biogenesis and bioenergetic functions at the BBB. Their results strongly confirmed the positive role of NRF2 in regulating the redox metabolic interplay that triggers the expression of antioxidative active elements and ultimately the protection of the BBB against OS damage [122]. NRF2 nuclear translocation and increased transcription of detoxifying enzymes and antioxidants effectively protect against chronic TS exposure [5]. However, they demonstrated impairment of NRF2 activity by chronic TS exposure, resulting in a suboptimal antioxidative response and consequent cellular OS damage, while acute exposure to TS and vapors from an electronic cigarette (e-Cig) initially enhances NRF2 expression and activation [11,85,122,123,154,155]. This facet of chronic TS and e-cig exposure and their effect on the NRF2–ARE system needs to be considered due to early-stage former smokers remaining at a high risk of developing cerebrovascular disorders for years after quitting [39]. Similar results have been observed in our recent work confirming TS/e-Cig induced cerebrovascular dysfunction and possibly other detrimental xenobiotics affects the BBB via oxidative stress [156] (see Figure 3).

## 9. Role of NRF2 in Hyperglycemia

Hyperglycemia or high blood sugar is described as an abnormally elevated rate of blood sugar (fasting levels of glucose > 130 mg/dL) and a sign of type 1 and type 2 diabetes [5]. Recent studies on the impact of hyperglycemia on NRF2 expression have revealed that while hyperglycemia does not directly affect NRF2 expression, it promotes its nuclear translocation [5,11]. It appears that both acute TS exposure and hyperglycemia significantly increase the nuclear/cytoplasmic NRF2 ratio and subsequent activation of antioxidant mechanisms. Indirectly, these data also suggest that TD2M-promoted and TS promote OS damage through similar mechanisms [8]. This is part of an acute response system activated in response to oxidative stress. In fact, recent studies have shown that inhibition of NLRP3 by NRF2 can improve diabetes-mediated cognitive and cerebrovascular impairments [157]. However, chronic persistence of hyperglycemia and/or exposure to TS impairs NRF2 activity leading to cerebrovascular damage and heighten the risk of neurological disorders. 

In spite of the observed facts such as enhanced TS-induced NRF2 activation by hyperglycemia (accounting for the existence of a cooperative effect) and enhanced-activation of endothelial pro-inflammatory responses by hyperglycemia, additional studies will be necessary to validate the underlying mechanisms and determine the corresponding pathophysiological implications relevant to the cerebrovascular system.

## 10. NRF2 Enhancers for the Treatment of Cerebrovascular Disorders: Repurposing of Antidiabetic Drugs

Recent studies have provided several dietary and therapeutic agents currently approved for the treatment of non-vascular and non-neurodegenerative pathologies that, on the contrary, do possess protective effects against the initiation/progression of neurodegenerative [1] and cerebrovascular diseases [5,122]. Metformin (MF), an oral anti-hyperglycemic agent, not only can enhance neurogenesis for the injured or degenerating neurovasculature [158], but it is also likely to diminish BBB disruption and decrease/inhibit ischemic injury upon stroke and impairment in neurodegenerative disorders [159,160,161,162,163]. MF has been shown to activate counteractive mechanisms which drastically reduce OS toxicity at the cerebrovascular and BBB levels such as those promoted by chronic TS exposure [83,122]. These beneficial effects are seemingly mediated by MF‘s activation of NRF2 [159] and include the suppression of TJ proteins downregulation and loss of BBB integrity by TS, the reduction of inflammation and oxidative stress, the renormalization of the expression levels of the major BBB glucose transporter Glut-1 and that of the anticoagulant factor thrombomodulin. Both AMPK-dependent and independent mechanisms are known to be involved in the mechanism of action of Metformin [164,165]. According to a study by Montalvo et al., treatment with Metformin resulted in an increased lifespan of aging mice brought about by the increase in antioxidant property and AMPK activation [166]. In addition, pre-treatment with MF has been discovered in distinct research to avoid ischemia-induced brain injury by activating the AMPK and NRF2 pathways and promoting rearrangement with ZO-1, occludin, and claudin-5 [159,160]. Recently our team showed that pretreatment with MF in vitro prevented downregulation of tight junction protein (ZO-1 and occludin) following chronic exposure to soluble cigarette smoke extract (sCSE). This effect was dependent upon activation of the NRF2 and AMPK pathways. Our study also revealed that that NRF2 activation (upregulation and translocation to the nucleus) is not exclusively dependent upon the activation of AMPK [122]. 

MF also has shown a neuroprotective effect on TS-induced cerebrovascular/BBB impairments to diminish the cerebrovascular toxicity accounting for a functional role of NRF2 and NRF2–ARE signaling pathways in protecting BBB integrity in chronically TS-exposed human BBB microvascular endothelial cells [11,85,122,123,159]. A recent study also demonstrated that treatment with MF in TS exposed mice restored levels of NRF2 and NQO1 to control levels in a dose-dependent manner. Along with these effects, levels of TJ proteins ZO-1 and Occludin were also restored [83]. In supporting the neuroprotective effect of anti-diabetic drug against oxidative stress in another study, Rosiglitazone (RSG) is a thiazolidinedione compound used for the treatment of TD2M that is well known to improve insulin resistance by regulating adiponectin gene expression [167]. Rosiglitazone is also considered as a transcription factor peroxisome proliferator-activated receptor (PPARγ) agonist [168,169]. Although the exact mechanism of action of rosiglitazone is not fully understood, recent studies have shown that this drug also possesses antioxidative features and can protect against OS damage and inhibit the inflammatory cascade through signaling inactivation by p38, JNK, and NF-κB [170,171,172]. Recently, Ceolotto et al. demonstrated that RSG protects endothelial cells against glucose-induced OS with an AMPK-dependent mechanism [156,168]. AMPK has been shown to promote NRF2 activity via nuclear accumulation further implying that RSG-mediated upregulation of PPARγ can be also associated with increased NRF2 activity [156]. In another study, Kadam et al. observed upregulation of NRF2 and its downstream target HO-1 as well as downregulation of toll-like receptor 4 (Tlr4) following RSG administration [173]. Activation of Tlr4 promotes NF-κB activity followed by pro-inflammatory cytokine production and stimulation of the innate immune system. A very recent study by our group has shown that RSG can seemingly mediate NRF2 upregulation and activation through upregulation of PPARγ expression [156]. Our data strongly suggest the possibility for a PPARγ-mediated mechanism of NRF2 upregulation/activation leading to the repairing of BBB integrity, decreased endothelial inflammatory responses as well as upregulation of NRF2 downstream signaling molecule NQO-1 which exert acute detoxification and cytoprotective functions [122]. Along this line, it is conceivable that other drugs presenting similar NRF2 enhancing effects (including nutritional dietary phytochemicals) could be repurposed for the treatment of OS and pro-inflammatory-dependent cerebrovascular and neurological disorders. However, more in vitro and in vivo studies will be necessary to confirm the feasibility and the beneficial effects of each treatment.

## 11. Conclusions

NRF2 plays a pivotal role in regulating redox homeostasis as well as the activation and modulation of antioxidant, anti-inflammatory, drug metabolism, detoxification, and radical scavenging functions [5,44]. Indeed, the NRF2–ARE signaling is significant for cytoprotection of cell survival against oxidative stress and preservation of the proper redox balance in cells and tissues by promoting antioxidative defenses neutralizing ROS and also blocking transcription of pro-inflammatory genes [174,175]. Nowadays, there is a growing research interest in investigating the cerebrovascular and neurodegenerative protective effect of NRF2 on maintaining the functional integrity of the BBB, preventing harmful CNS disorders, and the initiation/progression of neuroinflammatory and also the identification of novel approaches targeting NRF2 to prevent and/or reduce brain injury [14,88,125].

## Abbreviations

αSyn	Alpha-synuclein
AD	Alzheimer’s disease
ALS	Amyotrophic lateral sclerosis
AMPK	AMP-activated protein kinase
ARE	Anti-oxidant response element
BBB	Blood–brain barrier
BMVEC	Brain microvascular endothelial cells
CAT	Catalase
CNC	Cap‘n’collar
COPD	Chronic obstructive pulmonary disease
EGCG	Epigallocatechin gallate
Glut 1	Glucose transporter
GSH Px	Glutathione peroxidase
HD	Huntington’s disease
HO-1	Heme oxygenase-1
ICAM-1	Intercellular adhesion molecule-1
IKK	IκB kinase
KEAP1	Kelc-like ECH-associated protein 1
MF	Metformin
MS	Multiple sclerosis
NLRP3	Nod-like receptor protein 3
NQO-1	NAD(P)H: quinone reductase I
NTF2	Nuclear factor erythroid 2-related factor
PAMPs	Pathogen-associated molecular patterns
PD	Parkinson’s disease
PGC-1α	Proliferator-activated receptor gamma coactivator 1-alpha
PKC	Protein kinase C
PPARγ	Peroxisome proliferator-activated receptor gamma
PRR	Pattern recognition receptor
ROS	Reactive oxygen species
RSG	Rosiglitazone
SFN	Sulforaphane
SOD	Superoxide dismutase
TD2M	Type-2 diabetes mellitus
TBHQ	Terbutylhydroquinone
TBI	Traumatic brain injury
TEER	Trans-endothelial electrical resistance
TLRs	Toll-like receptors
TJ	Tight junction
TMCAO	Transient middle artery occlusion
TS	Tobacco smoking
Trx1	Thioredoxin-1
ZO-1	Zonulae occludentes-1
